# Burnout in Transition: A Qualitative Study of Nursing Interns’ Experiences and Implications for Clinical Management

**DOI:** 10.1155/jonm/6694491

**Published:** 2026-01-07

**Authors:** Shibo Zhang, Xia Zhang, Liu Zhang, Qiang Zhang, Huarong Liu, Linyu Li, Teng Huang, Yang He

**Affiliations:** ^1^ Women and Children’s Hospital of Chongqing Medical University, Chongqing, China

**Keywords:** burnout, clinical learning environment, clinical practicum, coping mechanisms, nursing interns, occupational stress, transition to practice

## Abstract

**Aim:**

To explore the experiences and perceptions of burnout among nursing interns during clinical practicum and to identify its underlying mechanisms and coping strategies, with implications for nursing management.

**Background:**

Nursing interns face considerable stress as they transition from classroom learning to clinical roles. Exposure to institutional demands, interpersonal tensions, and emotional exhaustion can lead to burnout, affecting their performance, well‐being, and professional identity.

**Methods:**

A descriptive qualitative study was employed. Twenty nursing interns who had experienced symptoms of burnout were recruited from three tertiary hospitals in western China. All participants were final‐year students in a registered nurse training program. Semistructured interviews were conducted, and data were analyzed using Braun and Clarke’s six‐phase thematic approach.

**Results:**

Four themes were developed through thematic analysis: (1) Violence under the system—interns experienced both explicit oppression (e.g., excessive workloads, rigid rules, and lack of voice) and implicit oppression (e.g., unpaid tasks, lack of respect, and normalized exploitation); (2) torn between idealism and reality—participants described idealized career expectations clashing with clinical realities, interpersonal conflicts, and uncertainty about professional values; (3) depletion of self‐resources—burnout manifested as physical exhaustion, emotional volatility, and cumulative effects such as emotional numbness and disengagement; and (4) finding their own path—interns coped through personal recharge strategies, self‐disclosure, quiet resistance, letting go of ambitious career goals, and, in some cases, resignation. These themes highlight the progressive, multidimensional nature of burnout and reveal both risks to interns’ well‐being and opportunities for targeted managerial interventions.

**Conclusions:**

Burnout among nursing interns is a dynamic and multifaceted process rooted in structural, emotional, and cognitive tensions. It impacts not only individual resilience but also long‐term workforce development and retention.

**Impact:**

Nursing managers should address both the structural and psychosocial stressors within clinical training environments. Interventions should include optimizing task allocation, building respectful mentorship systems, and providing emotional support services. Tailored strategies can enhance interns’ professional identity, strengthen clinical adaptation, and reduce the risk of premature burnout.

**Summary**

• What does this paper contribute to the wider global clinical community?
◦ Nursing interns are a critical but underexamined group within the global healthcare workforce. Their experiences of burnout during clinical practicum reveal unique structural and emotional challenges not addressed by current institutional support systems or assessment tools.
◦ Burnout among nursing interns reflects a multidimensional transition crisis—from idealistic professional aspirations to confronting systemic oppression, emotional depletion, and disengagement—highlighting the hidden costs of clinical education environments.
◦ Understanding the lived experiences of nursing interns contributes valuable insights into the design of intern‐specific interventions and organizational reforms, which are essential for strengthening professional identity, reducing early‐career attrition, and fostering a resilient global nursing workforce.

## 1. Introduction

Burnout has become a persistent and escalating occupational health issue across global healthcare systems, particularly affecting frontline medical personnel. More critically, it systematically undermines the quality of patient care by impairing clinical judgment, reducing work engagement, and increasing the likelihood of errors [1]. Recent global meta‐analyses suggest that over 30% [2] of nurses experience significant levels of burnout, with prevalence rising to 52.3% [3] or higher in high‐pressure environments such as emergency departments and intensive care units. These outcomes signal a broader crisis in workforce sustainability, manifesting in strained provider–patient relationships, elevated turnover rates, and diminished care quality.

To conceptualize this phenomenon, the Maslach burnout framework has been most widely applied [4]. It defines burnout as a three‐dimensional syndrome comprising emotional exhaustion (EE), depersonalization (DP), and reduced personal accomplishment (PA) [4]. EE refers to the depletion of mental and physical resources under sustained stress, often expressed as chronic fatigue, irritability, and feelings of being overwhelmed. DP reflects a defensive detachment from patients or colleagues, manifesting as cynicism, indifference, or treating others as impersonal objects. Reduced PA captures the decline in professional efficacy and fulfillment, when individuals perceive themselves as incompetent, ineffective, or unable to make meaningful contributions. Together, these dimensions provide a structured lens to understand how burnout develops and operates in healthcare contexts.

Nursing interns represent a critical yet underexplored subgroup within this framework. Positioned at the intersection of academic instruction and clinical practice, they are particularly vulnerable to emotional and cognitive strain. Challenges including heavy workloads, limited autonomy, role ambiguity, and strained interpersonal relationships are routinely encountered during clinical placements [5]. A systematic review revealed that burnout levels among nursing interns—initially lower than those of their nonmedical peers—tend to escalate rapidly throughout the practicum period, ultimately surpassing comparable groups in indicators such as EE and cognitive dysfunction [6]. Alarmingly, these experiences are associated with reduced professional identity, empathy fatigue, academic disengagement, and increased risk for substance use and suicidal ideation [7, 8].

While the Maslach framework captures the core dimensions of EE, DP, and reduced accomplishment, recent scholarship suggests that nursing interns also face unique stressors. Moral distress—arising when individuals are unable to act in accordance with their ethical beliefs due to organizational or systemic constraints—can be a significant source of burnout for nursing interns [9]. Repeated exposure to routine, low‐recognition tasks may also lead to a loss of professional meaning, eroding motivation and fulfillment [10], while the transition from student to practitioner can precipitate identity dissonance as interns struggle to reconcile their idealized self‐concept with the realities of clinical practice. Moreover, recent evidence shows that burnout in nurses not only compromises psychological well‐being but also manifests behaviorally as the rationing of nursing care, further undermining patient safety and care quality [11, 12]. Whether and how these dynamics emerge already during internship remains unclear.

Despite the widespread use of instruments such as the Maslach Burnout Inventory (MBI) [13] to assess burnout among healthcare trainees, recent international studies suggest that these tools may lack the sensitivity required to detect the specific stressors experienced by nursing interns. A multicountry psychometric study (Australia, the Netherlands, South Africa, and the United States) concluded that MBI may systematically underestimate burnout levels in this population due to its generalized structure and lack of clinical context alignment [14]. This raises concern over the adequacy of existing diagnostic measures and underscores the need for assessment tools that reflect the realities of early clinical training.

Efforts to alleviate burnout in nursing populations have included a range of interventions—mindfulness‐based stress reduction, resilience training, recreational therapy, and structured debriefing sessions among them [6]. However, a recent systematic review of 30 empirical studies found that these programs, while modestly effective for staff nurses, are rarely tailored to the needs of nursing interns, and often lack rigorous evaluation of long‐term outcomes [15]. This highlights a critical gap in workforce development strategies and calls for management‐led innovations that prioritize intern‐specific well‐being and retention.

Given these limitations in measurement and intervention, qualitative research provides an essential lens through which to explore the lived experiences and coping processes of nursing interns. Such methods are particularly effective in uncovering the emotional, structural, and social mechanisms that quantitative metrics may overlook. Emerging findings [16] suggest that even unconventional strategies—such as scheduled team breaks and peer reflection groups—can mitigate burnout symptoms, illustrating the value of grounding managerial interventions in firsthand accounts and contextual realities.

Accordingly, this study, guided by the Maslach burnout framework and informed by context‐specific factors, adopted a qualitative descriptive approach to explore the core experiences and underlying mechanisms of burnout among nursing interns, with the aim of providing evidence for the development of tailored assessment tools and management strategies. Specifically, the Maslach framework not only served as the theoretical foundation but also provided practical guidance for study design. Its three core dimensions—EE, DP, and reduced PA—were used to construct the semistructured interview guide, ensuring a systematic coverage of interns’ experiences across each dimension [4]. At the same time, the interview questions retained open‐ended flexibility, allowing participants to articulate context‐specific stressors beyond the framework, such as moral dilemmas, loss of meaning, and identity tensions, all of which are particularly salient during the internship stage [9, 12].

## 2. Methods

### 2.1. Study Design

This study employed a qualitative descriptive design to directly describe the burnout experiences of nursing interns, rather than deeply interpreting phenomena or building theories [17, 18]. Qualitative descriptive research is particularly suitable for exploring the lived experiences of specific groups in particular contexts while maintaining fidelity to the original data. For data analysis, we utilized Braun and Clarke’s [19] thematic analysis method as the specific analytical tool, which is compatible with qualitative descriptive design and facilitates the identification of patterns and construction of themes within the data. The study adhered to the Consolidated Criteria for Reporting Qualitative Research (COREQ) guidelines. The research team maintained reflexivity throughout the study by keeping reflective journals to document personal biases and presuppositions, enhancing methodological transparency. This study did not require formal ethics committee approval because it involved voluntary participation of adult students in noninterventional interviews without any clinical procedures or patient data. Participation was entirely anonymous and posed no potential harm. Written informed consent was obtained from all participants prior to data collection, and confidentiality was strictly maintained throughout the research process.

### 2.2. Setting and Participants

Participants were recruited through purposive sampling from three public tertiary hospitals in western China. Eligible participants were senior nursing students enrolled in a registered nurse training program, aged 18 years or above, and in the final year of their clinical placement. Participants were initially approached through internship coordinators, who distributed study invitations via internal email and hospital notice boards. This approach ensured that participation was entirely voluntary and that potential participants had sufficient time to consider the invitation before responding. The researchers had no supervisory or evaluative relationship with the students to minimize perceived coercion or power imbalance. To ensure the study focused on authentic burnout experiences, we adopted a multistage selection process involving clinical mentor, internship coordinator, and peer recommendations based on observed behaviors (e.g., persistent fatigue, decreased initiative, emotional fluctuation, and disengagement).

Prior to any interview activities, we formally administered the licensed Maslach Burnout Inventory–Human Services Survey (MBI‐HSS; authorized Chinese version) under research‐assistant supervision. In line with established cutoff scores, participants scoring ≥ 27 on EE, or ≥ 13 on DP, or ≤ 31 on PA were classified as “MBI‐positive” and progressed to the next screening step. MBI administration was used solely to establish a transparent eligibility gate and was not entered into a qualitative analysis. This study was conducted under a formal license from Mind Garden, ensuring compliant use of the MBI.

We selected the MBI despite its generic design because it remains the most widely validated and internationally recognized instrument for measuring burnout in healthcare populations. Using this standardized tool provided a transparent and comparable eligibility threshold, ensured methodological rigor, and allowed alignment with the existing literature. At the same time, we acknowledge that the MBI cannot fully capture the context‐specific burnout experiences of nursing interns. Therefore, it was used solely as an eligibility gate, while participants’ lived experiences were further explored and validated through structured screening interviews and in‐depth qualitative inquiry.

All recommended and MBI‐positive candidates underwent brief, structured, open‐ended interviews conducted separately by two independent researchers, each lasting approximately 5 min, to assess their recent experiences of sustained physical and EE, disengagement from tasks, feelings of helplessness, and doubts about the nursing profession. Only candidates who could clearly articulate such experiences with concrete examples, and who met the inclusion criteria (burnout symptoms persisting for at least 2 weeks, impairing academic or clinical performance, and causing significant subjective distress), were formally included—provided both evaluators independently agreed on their eligibility.

Additionally, we considered diversity in participants’ backgrounds, including gender, age, and clinical departments, to ensure a multifaceted perspective on burnout experiences. Through maximum variation sampling, we selected individuals with varied backgrounds from among those who met the initial screening criteria to enhance the breadth and depth of the data.

### 2.3. Data Collection

Prior to the interviews, participants completed a demographic questionnaire. The interview guide was developed based on the Maslach burnout framework and relevant literature and refined through three rounds of expert consultation (*n* = 5) by a panel of qualitative researchers, nursing educators, and clinical head nurses. A pilot phase with five participants helped further optimize the guide and procedure; these pilot interviews were excluded from the final analysis.

All interviews were conducted by researchers (first and second authors) trained in qualitative research methods, who had nursing education backgrounds but no direct teaching or evaluation relationships with the participants, minimizing power imbalances that might affect the interviews. Formal interviews were conducted between November 2024 and February 2025 in a quiet and private room at the researchers’ affiliated university. Before each interview, participants were reminded of the study’s purpose, confidentiality principles, and their right to withdraw, and written informed consent was obtained.

Interviews followed a semistructured format and lasted an average of 57 min (range: 48–66 min). All interviews were audio‐recorded, and field notes were taken by the interviewer to capture nonverbal cues and emotional responses. Interviewers used open‐ended and probing questions to encourage participants to share their experiences in depth (Supporting Information 1). After every five interviews, the research team held discussion meetings to assess data quality and thematic development. All interviews were audio‐recorded using a digital voice recorder (Sony ICD‐UX570F) with participants’ consent. The recordings were encrypted and stored on a password‐protected institutional drive accessible only to the research team.

Data collection continued until sufficient information power was achieved, meaning that the data set was judged to hold adequate depth, diversity, and relevance to address the study aim [20]. While earlier qualitative research commonly referred to “data saturation” as the point at which no new codes or themes emerge, we followed Braun and Clarke’s more recent argument that the notion of saturation is conceptually problematic and less applicable to reflexive thematic analysis [21]. Instead, our decision to conclude data collection was guided by an ongoing evaluation of information richness, adequacy, and the extent to which the data meaningfully supported our analytic and interpretive requirements. Through iterative team discussions, we determined that the existing interviews provided sufficient informational depth to fully explore the study’s objectives and theoretical framework. A total of 20 participants were included in the final analysis.

### 2.4. Data Analysis

Interview data were analyzed using Braun and Clarke’s [19] six‐phase reflexive thematic analysis, including (1) familiarization with the data, (2) generating initial codes, (3) searching for themes, (4) reviewing themes, (5) defining and naming themes, and (6) producing the final report. The coding process was primarily inductive, with themes actively constructed through iterative clustering and interpretation of codes, rather than passively emerging from the data. The Maslach burnout framework served as a sensitizing lens that informed both the interview design and the later interpretation, guiding attention to relevant dimensions of burnout without constraining an inductive analysis. This ensured that the findings remained grounded in participants’ narratives while connected to established theoretical constructs.

To illustrate the analytic pathway from raw data to final themes, including exemplar codes, subthemes, and supporting quotations, see Supporting Information 1.

Three researchers independently coded the transcripts, reconciled discrepancies through discussion, and refined a shared codebook. NVivo 12 software was used to organize transcripts, manage codes, compare coding outputs, and generate visual maps that supported theme development. To enhance credibility, the coding structure and thematic interpretation were reviewed by an external qualitative research expert, and member checking was conducted with selected participants to confirm the authenticity of the findings. Throughout the analysis, the team maintained memo‐writing and reflective journaling to ensure transparency and traceability. Final themes were established through consensus and supported with direct participant quotations.

### 2.5. Rigor and Trustworthiness

To ensure the rigor of this study, we evaluated our processes based on four key criteria: credibility, confirmability, dependability, and transferability [22].

Credibility was enhanced through multiple strategies: First, the research team included two nurse educators and one nursing education administrator, all possessing strong contextual knowledge of the clinical learning environment. Second, all team members received formal training in qualitative research methodologies before the study and were supervised by an experienced qualitative researcher. Third, the interview guide was developed through a literature‐informed process and reviewed by an expert panel (*n* = 5) to ensure appropriate question design. Fourth, we employed triangulation by having multiple researchers independently analyze the data, comparing field notes with interview records. Finally, member checking was conducted with eight participants who reviewed thematic summaries and selected quotations to verify whether our interpretations aligned with their experiences. Participant feedback was incorporated into the final analysis; for example, based on participant suggestions, we modified the wording of the “Torn between idealism and reality” theme to more accurately reflect their experiences.

Confirmability was achieved through having three researchers independently analyze the data and finalizing the findings through group consensus. We maintained a detailed audit trail documenting all analytical decisions, coding changes, and thematic refinements throughout the study. Additionally, each researcher explicitly recorded their presuppositions about nursing internship experiences before analysis and reflected on how these might influence interpretation throughout the research process. For instance, one researcher had previously served as a nursing internship instructor and held inherent views about internship structures; the team paid special attention to and discussed potential interpretive biases arising from these experiences.

Dependability was supported by directly quoting participants to substantiate key findings, ensuring that interpretations were clearly grounded in the data. We created a detailed codebook containing definitions, examples, and usage rules for each code to maintain coding consistency. Furthermore, we held regular coding consistency check meetings, calculating intercoder agreement rates (averaging 83%). When interpretations differed, consensus was reached by returning to the original data and discussing different perspectives.

Transferability was supported by providing detailed descriptions of the study’s context, sampling strategy, data collection, and analysis procedures. We documented specific characteristics of the nursing internship system in tertiary hospitals in western China, helping readers assess the applicability of the findings to similar environments. Additionally, we provided rich participant background information and thick description, including direct quotations and contextual descriptions, enabling readers to evaluate whether the findings apply to other situations. To minimize selection bias associated with a standardized eligibility gate, we combined MBI‐based eligibility with a brief structured screening and independent dual adjudication, and we maintained an audit trail for all inclusion decisions.

## 3. Results

A total of 20 nursing interns were included in this study. All participants were final‐year students in a registered nurse training program and were approaching the completion of their clinical practicum. The participants ranged in age from 21 to 23 years, with a mean age of 21.8 ± 0.77 years. The sample consisted of 18 females and 2 males. Demographic details are presented in Table 1.

**Table 1:** Demographic characteristics and burnout scores of participants (*n* = 20).

Participant	Age	Gender	Internship duration (months)	MBI		
				EE	DP	PA
1	21	Female	10	22	10	30
2	22	Female	12	20	12	28
3	21	Female	10	25	14	27
4	22	Female	11	19	11	29
5	21	Female	10	23	13	26
6	22	Female	12	18	10	30
7	21	Female	11	21	9	31
8	23	Female	12	20	12	28
9	22	Male	10	22	11	29
10	21	Female	10	25	14	27
11	22	Female	10	19	10	30
12	23	Female	12	22	9	26
13	21	Female	10	24	13	26
14	22	Female	11	20	11	29
15	21	Female	11	21	9	31
16	23	Female	12	22	10	30
17	22	Male	10	19	12	28
18	21	Female	12	23	14	27
19	22	Female	10	18	10	30
20	23	Female	12	20	12	28

Abbreviations: DP, depersonalization; EE, emotional exhaustion; PA, personal accomplishment.

This study sought to explore the lived experience of burnout among nursing interns during clinical practicum. Initial coding of the interview data generated 15 conceptual codes that reflected various psychological, interpersonal, and structural aspects of the burnout process. Through an iterative process of analysis and thematic refinement, 4 interrelated themes were identified: (1) Violence under the system, (2) Torn between idealism and reality, (3) Depletion of self‐resources, and (4) Finding their own path. These themes collectively captured the progressive, multidimensional nature of burnout and how interns made sense of and responded to it across their training trajectory.

### 3.1. Violence Under the System

During clinical internships, nursing interns are often subjected to multifaceted pressures arising from institutional structures, entrenched hierarchical culture, and administrative management. These pressures are not only manifested in heavy workloads but also in hidden forms of exploitation, neglect, and inequitable treatment embedded in the clinical environment. The violence they experience can be categorized into explicit oppression and implicit oppression, each reflecting a different but complementary mode of systemic harm that erodes interns’ physical, emotional, and professional well‐being.

#### 3.1.1. Explicit Oppression

Explicit oppression refers to direct, tangible, and observable stressors encountered by nursing interns. These typically manifest as long and inflexible working hours, repetitive basic tasks that limit learning opportunities, and rigid managerial rules that leave no room for negotiation. Such oppression originates from institutionalized expectations that interns should endure hardship unquestioningly as part of their training, perpetuating a cycle of overwork and disempowerment.

“Clinical work is exhausting—we often work ten hours straight without even a lunch break.” (Participant 3)

*“Every single day, it’s just checking blood pressure and taking temperatures over and over again.”* (Participant 14)

Interns are also expected to rapidly master complex theoretical knowledge while preparing for frequent assessments and skill evaluations.

*“There’s just so much to learn, and I’ve never seen this material before—yet I have an exam next week.”* (Participant 1)

A lack of voice and decision‐making power also constitutes a clear form of oppression. Interns’ suggestions are routinely dismissed, and they are often unable to decline or even question unreasonable assignments.

*“They never listen to us. Every time there’s a meeting, we’re asked to leave the room.”* (Participant 15)

*“I once made a* suggestion *to the head nurse, but it didn’t make any difference.”* (Participant 7)

Emotional pressure from supervisors and patients further compounds this form of oppression. Interns are often held to high standards by senior staff and may be reprimanded or even humiliated for mistakes.

*“Some instructors get visibly angry when I’m not skilled enough—they even say things like, ‘How can you not do something this simple?’”* (Participant 8)

#### 3.1.2. Implicit Oppression

Implicit oppression consists of subtler yet equally detrimental stressors that are embedded in the organizational culture and social interactions. These include unpaid labor disguised as “learning opportunities,” chronic lack of support from mentors or colleagues, and repeated experiences of being disrespected or treated as inferior. Such dynamics are often normalized through the rhetoric of “tradition” and “rite of passage.”

*“When I first joined the department, the instructor demonstrated one task, then left me to handle everything on* my *own for the entire unit.”* (Participant 7)

Such forms of exploitation are often normalized through departmental culture, justified under the pretense of “tradition.”

*“Our instructors said they had to do everything themselves when they were interns—so we shouldn’t expect anything different.”* (Participant 2)

Even after clocking out, interns are often burdened with additional tasks, including those unrelated to their training.

*“After work, I still have to help my instructor with PowerPoint slides.”* (Participant 6)

Interns are also subject to inappropriate demands or disrespect from patients, which can undermine their professional identity and sense of dignity.

*“A patient who* could *take care of himself just lay there and said, ‘Hey little nurse, go fetch my takeout.’”* (Participant 1)

Due to their low status in the clinical hierarchy, interns are also vulnerable to being ordered around by staff who are not their designated mentors.

*“She wasn’t even my supervisor, yet she ordered me to do things for her. It made me feel completely disrespected.”* (Participant 11)

These dynamics resonate with Bourdieu’s concept of symbolic violence, where unequal power relations are disguised as tradition, discipline, or “professional training,” and thus come to be perceived as natural and legitimate. In this context, nursing interns internalize institutional hierarchies and normalize unfair treatment, often blaming themselves for being “inadequate” rather than questioning the structural conditions that constrain them. The rhetoric of “rite of passage” serves to reproduce compliance, ensuring that exploitation is transmitted from one generation of nurses to the next. Consequently, systemic harm becomes invisible, embedded not only in explicit managerial rules but also in the cultural fabric of the clinical environment. This hidden dimension of violence erodes interns’ sense of agency, dignity, and professional identity while reinforcing their marginal position within the healthcare hierarchy.

### 3.2. Torn Between Idealism and Reality

Nursing interns often experience a profound tension between their idealized vision of nursing and the demanding realities of clinical work. This theme captures a gradual process of cognitive disillusionment, in which enthusiasm, moral aspiration, and professional ideals are progressively eroded by repetitive tasks, limited autonomy, and a culture that undervalues interns’ contributions.

At the beginning of their internships, many viewed clinical practice as a stage for demonstrating competence and fulfilling their vocational ideals: *“I was really looking forward to proving myself in the clinical setting.” (Participant 20)*

However, their early optimism soon collided with repetitive and trivial tasks that offered little learning value. As one participant put it, *“Most of my time is spent checking vital signs. I thought work should be meaningful and rewarding—not just measuring blood pressure all day.” (Participant 6).* These accounts reveal how narrow role boundaries and repetitive routines restricted opportunities for growth, transforming initial excitement into disappointment and self‐doubt.

This emotional tension deepened through interpersonal encounters. Harsh criticism from supervisors or rejection from patients often undermined interns’ confidence and professional dignity.

“I made a small mistake, and my mentor scolded me loudly in front of the patient—it was incredibly *embarrassing*.” (Participant 17)

“When I was about to give an injection, the patient saw my intern badge and refused to let me do it.” (*Participant* 15)

Such moments of humiliation and exclusion reinforced feelings of marginalization and helplessness. Interns began to internalize these experiences as personal inadequacy rather than systemic issues, reflecting how hierarchical power relations shape emotional responses in clinical learning environments.

As disillusionment accumulated, interns increasingly questioned whether nursing could provide the sense of purpose and respect they once imagined. This conflict represents a form of identity dissonance—where the “aspired self,” grounded in moral idealism, clashes with the “experienced self,” constrained by institutional realities. Some coped by lowering expectations, emotionally withdrawing, or adopting a pragmatic stance of “just getting through the day.”

Ultimately, this erosion of idealism marks a pivotal point in the burnout trajectory: Moral commitment gives way to guarded survival, and the initial dream of nursing as a calling transforms into a struggle for endurance within a rigid system.

### 3.3. Depletion of Self‐Resources

As clinical demands intensified, nursing interns described a steady depletion of both physical and emotional reserves. This theme illustrates how prolonged workload, emotional suppression, and moral strain accumulate into a state of profound exhaustion, blunting empathy and motivation over time.

At first, exhaustion was primarily physical. Long hours of standing, lifting patients, and continuous multitasking left many feeling chronically fatigued and unable to recover between shifts.

“Walking around and lifting patients all day has left me with constant pain in my back and shoulders.” (Participant 8)

However, physical fatigue soon intertwined with emotional strain. Interns learned to suppress distress in front of patients and mentors, maintaining composure despite frustration or fear. Yet, once alone, these suppressed emotions erupted:

“A patient yelled at me, and I held it together. But as soon as I walked out of the room, I burst into tears.” (Participant 9)

Over time, the persistent tension between endurance and suppression transformed exhaustion into emotional numbness. Interns reported feeling detached and mechanical in their interactions:

“When patients talk about their symptoms, I just nod mechanically. I don’t feel anything inside.” (Participant 6)

This progressive desensitization represents more than simple tiredness—it signals empathic fatigue, a defensive adaptation to chronic stress that protects individuals from further emotional overload while eroding the very relational core of nursing. As compassion gives way to detachment, interns shift from active engagement to passive survival, marking the deep psychological stage of burnout.

### 3.4. Finding Their Own Path

Despite pervasive exhaustion and disillusionment, many interns sought ways to regain agency and rebuild psychological balance. This theme depicts the spectrum of coping strategies—from active self‐care to quiet resistance and, for some, eventual disengagement—through which interns attempted to preserve dignity and control within constraining clinical systems.

Some adopted active coping behaviors aimed at emotional recovery. Physical exercise, leisure activities, and social sharing were described as brief sanctuaries that helped them “breathe again.” These efforts offered temporary relief and a sense of normalcy, reminding interns of identities beyond their work roles.

“I exercise every day. It really helps me unwind.” (Participant 5)

“Sometimes I talk things through with my classmates—we encourage and help each other.” (Participant 16)

As burnout deepened, coping gradually shifted from engagement to avoidance. Several interns admitted to procrastinating, simplifying procedures, or emotionally detaching to conserve limited energy. Such quiet resistance—often expressed as subtle noncompliance rather than confrontation—became a way to reclaim small pockets of autonomy. These behaviors, while adaptive in the short term, also signaled growing disengagement from professional ideals.

For others, prolonged strain triggered a reevaluation of career trajectories. Some deliberately scaled back ambitions or sought less demanding positions that promised stability and personal time:

“At this point, I just want to be a regular nurse. I’m not chasing promotions anymore.” (Participant 20)

A few reached the endpoint of withdrawal, choosing to leave the profession altogether:

“I really tried to like this job, but the more I do it, the more I realize this isn’t the life I want.” (Participant 2)

Taken together, these narratives reveal coping as a dynamic continuum rather than a single act—shifting from restorative efforts to self‐protective withdrawal and, ultimately, to exit when personal values and systemic constraints become irreconcilable. This adaptive progression underscores both the resilience and the vulnerability of nursing interns navigating burnout within rigid institutional contexts.

## 4. Discussion

Our study indicates that burnout among nursing interns is not primarily a consequence of individual resilience deficits but results from intertwined explicit and implicit systemic and cultural pressures—including heavy workload, limited autonomy, clinical culture, and feelings of marginalization. Within China’s strict hierarchy and performance‐driven training system, the narrative that “success equals conformity and performance” is reinforced, while failure is stigmatized as incompetence, increasing psychological burden. In a study of 972 Chinese nursing interns, 97.8% experienced moderate burnout and 55.3% reported secondary traumatic stress [23]. In contrast, a global systematic review reported an overall burnout prevalence of approximately 46% among nursing students—47.7% moderate and 22.5% severe [24]. Another systematic review covering 12 European countries found that only nine nations had burnout rates above 25%, with a maximum of 78%. These contrasts vividly demonstrate how the structural hierarchy and lower levels of intern engagement in Western settings may buffer against burnout, while China’s institutional and performance‐driven culture exerts uniquely profound impacts on interns’ mental well‐being [25]. Beyond these prevalence contrasts, it is important to recognize how the weight of institutional authority and the framing of interns as “subordinates,” rather than learners, condition the burnout trajectory. Limited autonomy, the normalization of silence, and the expectation of unquestioned compliance amplify EE and accelerate detachment. These sociocultural mechanisms illustrate that burnout among Chinese nursing interns is not only a psychological response to workload but also a reflection of the broader cultural and organizational environment in which they are embedded.

The study indicates that burnout among nursing interns develops progressively, aligning with Maslach’s three‐dimensional model of burnout [4]. Initially, interns experience EE—marked by mood swings and mild physical discomfort—primarily driven by excessive workloads, role ambiguity, and moral distress arising from ethically challenging situations where they feel unable to act in patients’ best interests due to institutional constraints [26]. This often evolves into DP, where interns become increasingly detached from patients and colleagues, reflecting both emotional fatigue and diminished student agency—the sense of autonomy and ability to influence outcomes [27]. Ultimately, as the gap widens between idealized career expectations and clinical realities, interns exhibit reduced PA, with weakened professional identity and self‐efficacy, echoing the challenges described in the professional identity formation literature [28]. Furthermore, Yi [29] found that burnout levels tend to increase with each academic term, creating a vicious cycle of “stress–burnout–declining efficacy” that profoundly affects both clinical experiences and future career development.

Nursing interns commonly adopt emotion‐focused coping strategies—such as venting, peer support, and social media expression—to relieve stress and burnout. Although these approaches offer short‐term relief, they fail to address systemic drivers of burnout, including understaffing, overwhelming workloads, and insufficient institutional support [30]. Consequently, some interns exhibit “defensive disengagement,” characterized by reduced enthusiasm, streamlined clinical routines, and lowered career aspirations, reflecting deterioration in professional identity. In more severe cases, prolonged stress and disillusionment lead a small number of interns to resign entirely from the profession, choosing to pursue alternative careers that align better with their values and well‐being [31]. This phenomenon of resignation deserves more attention, as it not only represents the endpoint of the burnout trajectory but also undermines workforce retention and exacerbates early‐career attrition in nursing [32]. Interventions that emphasize individual resilience while neglecting organizational reform have shown limited long‐term impact [33]. Additionally, negative workplace climates and the absence of structured support systems further exacerbate burnout risk [34]. Addressing this issue requires institutions to take structural responsibility, shifting beyond individual‐focused solutions toward integrated, multilevel support systems that strengthen interns’ adaptive capacity and restore professional commitment.

Based on our findings, we believe that nursing managers should address intern burnout through both structural reforms and cultural changes [35]. Specifically, we propose the following actionable recommendations. First, develop and disseminate a written task guideline that clearly defines appropriate, learning‐oriented duties and explicitly prohibits repetitive, noneducational assignments, with regular audits to ensure adherence [36]. Second, establish biweekly structured feedback sessions between mentors and interns, supported by standardized forms to document progress, adjust expectations, and collaboratively set realistic learning goals [37]. Third, implement a phased intervention program that combines weekly mindfulness exercises, monthly stress‐management workshops, and one‐on‐one cognitive reframing coaching, which has demonstrated high acceptability and engagement over time [38]. Similar structured resilience and coping interventions have demonstrated effectiveness in high‐stress settings such as intensive care units [39], suggesting their potential applicability to nursing interns as well. Nevertheless, the feasibility of these interventions in resource‐limited or high‐turnover environments may be constrained by time, staffing, and financial pressures. To address this, low‐cost adaptations—such as incorporating short mindfulness practices into morning briefings, using digital platforms (e.g., WeChat groups) for peer reflection or integrating feedback into routine handovers—could provide more realistic avenues for implementation without overburdening clinical departments [40–42].

This study offers a nuanced understanding of how burnout develops among nursing interns, highlighting its progressive and multifaceted nature. To address this, future interventions should adopt an integrated approach that combines institutional reform, educational support, emotional regulation, and personal development. Some studies have underscored the value of multilayered support systems in fostering adaptability and reducing burnout [43]. As Maslach argues, lasting change requires a shift from individual coping strategies to systemic organizational transformation—addressing the structural roots of burnout is essential to sustaining a resilient healthcare workforce [4]. International evidence also offers valuable lessons: For instance, the UK’s Preceptorship Programs emphasize structured mentor–intern relationships and have reduced early‐career attrition [44], Australian hospitals have reported success with structured peer‐debriefing groups that mitigate EE [45], and several Nordic countries have developed intern well‐being hubs providing confidential counseling and peer support [46]. Such programs illustrate how multilevel strategies can be tailored to different contexts and may serve as useful models for adapting interventions to the Chinese healthcare system.

Beyond addressing burnout through structural and cultural reforms, our findings also highlight the need for context‐specific assessment tools tailored to nursing interns. Existing instruments, such as the MBI, fail to capture key dimensions of interns’ unique experiences, including implicit exploitation, diminished professional respect, misalignment between clinical tasks and educational objectives, and the availability of institutional and emotional support. Future tools should include indicators that assess these nuanced stressors, integrating both quantitative scales and qualitative feedback to reflect the multidimensional nature of burnout in this population. For example, subscales measuring perceived respect and recognition, clarity of role boundaries, frequency of noneducational tasks, and accessibility of mentorship or counseling services could improve sensitivity and diagnostic accuracy. Developing and validating such tools can help nursing managers identify at‐risk interns earlier and implement more targeted, effective interventions to mitigate burnout.

## 5. Conclusion

This study underscores that burnout among nursing interns is not simply an issue of individual resilience, but the result of a complex interplay between systemic pressures, unmet professional ideals, and the depletion of personal resources. Both overt demands—such as excessive workloads and rigid management—and covert exploitation—such as noneducational, unpaid, or repetitive tasks—contribute to emotional fatigue and weaken professional identity. These impacts are compounded by structural flaws in the healthcare system and insufficient educational support, resulting in progressive EE and disengagement. Addressing this issue requires a shift from focusing solely on individual coping strategies to implementing broader structural and cultural changes.

To reduce burnout, nursing management should establish clear role definitions and minimize noninstructional duties for interns, ensuring that assignments align with learning objectives. Emotional support mechanisms—such as peer‐reflection groups, confidential counseling services, and mindfulness‐based workshops—can help interns manage emotional volatility and build resilience. Structured feedback mechanisms, including regular mentor–intern meetings and anonymous suggestion channels, can facilitate open communication and expectation adjustment. In addition, career development initiatives such as mentor pairing and public recognition of achievements may enhance interns’ professional identity and support sustainable career development. Moving forward, the development of context‐specific assessment tools and validated, multilevel intervention strategies—grounded in longitudinal and mixed‐method research—are essential to safeguarding interns’ mental health and facilitating their transition into the nursing workforce.

Future research should also focus on developing context‐specific assessment tools for nursing interns. Such instruments need to capture unique stressors—such as implicit exploitation, unclear role boundaries, and limited institutional support—that existing tools such as the MBI may overlook. Validated, intern‐specific tools would enable earlier identification of at‐risk individuals and facilitate more targeted interventions.

## 6. Limitations

This study has certain limitations. The findings are shaped by the specific context of tertiary hospitals in western China, and readers may consider their transferability to other settings with similar characteristics. The predominance of female participants may also reflect gendered emotional experiences and coping styles. In addition, self‐selection bias cannot be ruled out, as more reflective or articulate interns may have been more willing to participate. These factors highlight the need for more diverse and inclusive samples in future research to explore how contextual and individual differences shape nursing interns’ experiences of burnout.

## Conflicts of Interest

The authors declare no conflicts of interest.

## Author Contributions

Shibo Zhang: conceptualization, methodology, data collection, data analysis, drafting of the manuscript, and critical revision.

Xia Zhang: methodology, coding verification, data analysis, and manuscript revision.

Liu Zhang: data verification, refinement of thematic analysis, literature review updates, and contribution to manuscript editing during the revision stage.

Qiang Zhang: methodological guidance, supervision of research procedures, and manuscript review.

Huarong Liu: clinical expertise, interpretation of results, and revision of manuscript content.

Linyu Li: coding reliability checking, audit trail development, and support in data management.

Teng Huang: supervision, project administration, and corresponding author responsibilities.

Yang He: supervision, methodological oversight, interpretation of findings, and corresponding author responsibilities.

Shibo Zhang and Xia Zhang have contributed equally to this work and share first authorship. Teng Huang and Yang He share corresponding authorship.

## Funding

This study was supported by the Medical Research Project of Chongqing Health Commission (2025WSJK069) and the Educational and Teaching Reform Project of Nursing College of Chongqing Medical University (20250210).

## Supporting Information

This study includes supporting information which provides detailed interview guides, the thematic analysis coding table, and raw data records. These materials support the primary findings and provide further context to the analysis. The interview guide outlines the key questions used to explore the experiences of burnout among nursing interns, while the coding table illustrates the thematic analysis process. Raw data records contain anonymized excerpts from participants’ interviews that were used to generate the study’s themes.

## Supporting information


**Supporting Information** Additional supporting information can be found online in the Supporting Information section.

## Data Availability

The data that support the findings of this study are available from the corresponding authors upon reasonable request.
