# Supplementary material for: Burnout in Transition: A Qualitative Study of Nursing Interns’ Experiences and Implications for Clinical Management
Source: J Nurs Manag. 2026 Jan 7;2026:6694491. doi: 10.1155/jonm/6694491 (PMC12776595; doi:10.1155/jonm/6694491)
Supplement: Supplementary file 1 — Supporting Information Additional supporting information can be found online in the Supporting Information section. [file JONM-2026-6694491-s001.docx]

| Table S1: Demographic Characteristics of Participants |
| --- |
| What situations during your internship caused you stress or anxiety? What were the main sources (e.g., clinical tasks, communication, assessments, teamwork)? |
| How do you understand the concept of "burnout"? Do you think your internship experience fits this description? |
| Which aspects of your internship drained your energy the most? Why? |
| What emotional or behavioral changes did you notice when feeling burned out? |
| Has burnout affected your physical health (e.g., insomnia, fatigue)? |
| How has burnout influenced your academic performance, internship behavior, or daily life? |
| What do you think are the main causes of your burnout? |
| How do you usually cope with burnout? Are these strategies effective? |
| Has this experience influenced your view of the nursing profession or your career plans? |

| Table S2: Coding Framework for Thematic Analysis | | | |
| --- | --- | --- | --- |
| Theme (Level 1) | Sub-theme (Level 2) | Category (Level 3) | Exemplary Quote |
| Violence under the System | Explicit Oppression | Long working hours, no break | “Clinical work is exhausting—we often work ten hours straight without even a lunch break.” (Participant 3) |
|  |  | Repetitive basic tasks | “Every single day, it’s just checking blood pressure and taking temperatures over and over again.” (Participant 14) |
|  |  | Learning and exam pressure | “There’s just so much to learn, and I’ve never seen this material before—yet I have an exam next week.” (Participant 1) |
|  |  | Lack of voice | “They never listen to us. Every time there’s a meeting, we’re asked to leave the room.” (Participant 15) |
|  |  | Suggestions dismissed | “I once made a suggestion to the head nurse, but it didn’t make any difference.” (Participant 7) |
|  |  | Public scolding and humiliation | “Some instructors get visibly angry when I’m not skilled enough—they even say things like, ‘How can you not do something this simple?’” (Participant 8) |
|  | Implicit Oppression | Exploitation disguised as learning | “When I first joined the department, the instructor demonstrated one task, then left me to handle everything on my own for the entire unit.” (Participant 7) |
|  |  | Cultural oppression | “Our instructors said they had to do everything themselves when they were interns—so we shouldn’t expect anything different.” (Participant 2) |
|  |  | Tasks after work | “After work, I still have to help my instructor with PowerPoint slides.” (Participant 6) |
|  |  | Disrespect from patients | “A patient who could take care of himself just lay there and said, ‘Hey little nurse, go fetch my takeout.’” (Participant 1) |
|  |  | Ordered by non-supervisors | “She wasn’t even my supervisor, yet she ordered me to do things for her. It made me feel completely disrespected.” (Participant 11) |
| Torn Between Idealism and Reality | Idealized Career Expectations | Eager to prove oneself | “I was really looking forward to proving myself in the clinical setting.” (Participant 20) |
|  |  | Disillusioned with work | “Most of my time is spent checking vital signs. I thought work should be meaningful and rewarding—not just measuring blood pressure all day.” (Participant 6) |
|  |  | Skill-reality gap | “I thought it would be a simple task, but when I try to do it, my hands just don’t cooperate.” (Participant 18) |
|  |  | Disappointment in the profession’s sacredness | “I used to think nursing was a noble profession, but after entering the clinic, I realized that idea is almost laughable.” (Participant 9) |
|  |  | Life disrupted | “I thought I’d have more free time during my internship. Now I just want to collapse in bed when I get home.” (Participant 2) |
|  |  | Effort-reward imbalance | “I thought nursing was a secure and reliable career, but now I see nurses working constant overtime for far less pay than I expected.” (Participant 14) |
|  | Interpersonal Conflicts | Criticized publicly by superiors | “I made a small mistake, and my mentor scolded me loudly in front of the patient. It was incredibly embarrassing.” (Participant 17) |
|  |  | Lack of patient trust | “When I was about to give an injection, the patient saw my intern badge and refused to let me do it.” (Participant 15) |
|  |  | Judgment ignored | “I noticed a patient’s blood pressure was high and reported it, but the doctor just said, ‘Keep monitoring,’ without any further explanation.” (Participant 11) |
|  |  | Peer pressure | “There’s someone in my group who picks up every skill immediately. I keep struggling in comparison.” (Participant 2) |
| Depletion of Self-Resources | Physical Exhaustion | Body pain, insomnia | “Walking around and lifting patients all day has left me with constant pain in my back and shoulders.” (Participant 8) |
|  |  | Physical overwork | “The work during the day is so intense that I often can’t sleep at night from sheer exhaustion.” (Participant 1) |
|  | Emotional Volatility | Suppressing emotions, outbursts later | “A patient yelled at me, and I held it together. But as soon as I walked out of the room, I burst into tears.” (Participant 9) |
|  |  | Emotions spill into private life | “Back in the dorm, I snap at people over the tiniest things… I know it’s wrong, but I can’t control it.” (Participant 20) |
|  | Cumulative Effects | Emotional numbness | “When patients talk about their symptoms, I just nod mechanically. I don’t feel anything inside.” (Participant 6) |
|  |  | From passion to indifference | “The excitement I had at the beginning of my internship is long gone. Now I just want to get through my tasks.” (Participant 3) |
|  |  | Desensitized to criticism | “I used to feel so guilty when my mentor criticized me. Now all I think is, ‘Just get it over with.’” (Participant 8) |
|  |  | Aversion, irritability | “Every time I think about going to work, I feel inexplicably agitated—I don’t even know why.” (Participant 4) |
| Finding Their Own Path | Personal Recharge Strategies | Exercise, leisure, outdoors | “I exercise every day. It really helps me unwind.” (Participant 5) |
|  |  |  | “I treat myself to a big meal and karaoke when I get a break.” (Participant 7) |
|  |  |  | “After a stressful day, I like to take a walk in the park. The fresh air really calms me down.” (Participant 12) |
|  | Self-Disclosure | Sharing with family and peers | “When I get home from work, I always talk to my parents about everything that happened in the hospital.” (Participant 9) |
|  |  | Anonymous venting online | “Every night I anonymously write about my day online and keep refreshing to read the replies.” (Participant 18) |
|  |  | Psychological distancing | “I imitate the show Severance—I switch to another persona after work and try to forget everything that happened on the job.” (Participant 7) |
|  | Quiet Resistance | Procrastination, avoidance | “I pretended to agree, but deep down I didn’t want to do it. I figured someone else would step in, so I just kept stalling.” (Participant 3) |
|  |  | Simplifying procedures | “Sometimes I intentionally skip parts of the process. They don’t seem meaningful, so why waste time?” (Participant 15) |
|  | Letting Go of Goals | Lowering standards, giving up graduate school | “I used to want to pursue graduate school, but now I don’t think it’s worth it. The pressure is too much, and there’s no guarantee it’ll lead to a better future.” (Participant 13) |
|  |  | Choosing low-pressure jobs | “I’m planning to work at a township health center. The pressure is lower, life is more stable—it seems like a good option.” (Participant 20) |
|  | Resignation | Changing careers, leaving nursing | “After graduation, I’ll probably change careers altogether—start a new chapter in life.” (Participant 12) |
|  |  | Disillusionment with profession | “I really tried to like this job, but the more I do it, the more I realize this isn’t the life I want.” (Participant 2) |
|  |  | Economic reasons | “The pay is too low for how much work and stress we have. I started to think about switching to a job with better pay and more regular hours.” (Participant 17) |
